# An investigation of Hebbian phase sequences as assembly graphs

**DOI:** 10.3389/fncir.2014.00034

**Published:** 2014-04-08

**Authors:** Daniel G. Almeida-Filho, Vitor Lopes-dos-Santos, Nivaldo A. P. Vasconcelos, José G. V. Miranda, Adriano B. L. Tort, Sidarta Ribeiro

**Affiliations:** ^1^Brain Institute, Federal University of Rio Grande do NorteNatal, Brazil; ^2^Circuit Dynamics and Computation Laboratory, Champalimaud Neuroscience ProgrammeLisbon, Portugal; ^3^Universitary Center of Rio Grande do NorteNatal, Brazil; ^4^Physics Department, Federal University of BahiaSalvador, Brazil

**Keywords:** cell assembly, phase sequence, graph, sleep, learning and memory

## Abstract

Hebb proposed that synapses between neurons that fire synchronously are strengthened, forming cell assemblies and phase sequences. The former, on a shorter scale, are ensembles of synchronized cells that function transiently as a closed processing system; the latter, on a larger scale, correspond to the sequential activation of cell assemblies able to represent percepts and behaviors. Nowadays, the recording of large neuronal populations allows for the detection of multiple cell assemblies. Within Hebb's theory, the next logical step is the analysis of phase sequences. Here we detected phase sequences as consecutive assembly activation patterns, and then analyzed their graph attributes in relation to behavior. We investigated action potentials recorded from the adult rat hippocampus and neocortex before, during and after novel object exploration (experimental periods). Within assembly graphs, each assembly corresponded to a node, and each edge corresponded to the temporal sequence of consecutive node activations. The sum of all assembly activations was proportional to firing rates, but the activity of individual assemblies was not. Assembly repertoire was stable across experimental periods, suggesting that novel experience does not create new assemblies in the adult rat. Assembly graph attributes, on the other hand, varied significantly across behavioral states and experimental periods, and were separable enough to correctly classify experimental periods (Naïve Bayes classifier; maximum AUROCs ranging from 0.55 to 0.99) and behavioral states (waking, slow wave sleep, and rapid eye movement sleep; maximum AUROCs ranging from 0.64 to 0.98). Our findings agree with Hebb's view that assemblies correspond to primitive building blocks of representation, nearly unchanged in the adult, while phase sequences are labile across behavioral states and change after novel experience. The results are compatible with a role for phase sequences in behavior and cognition.

## Introduction

The firing synchronization of groups of neurons is a well-known phenomenon in the brain (Harris et al., 2003; Buzsáki, 2004; Harris, 2005; Canolty et al., 2010; Lopes-dos-Santos et al., 2011). According to the cell assembly hypothesis (Hebb, 1949), neurons transiently synchronize in order to form elementary units of information processing. Some reports have provided experimental evidence that assembly activity, i.e., the co-firing of assembly members, can be related to formation of memories and behavior (Wilson and McNaughton, 1994; Stopfer et al., 1997; Robbe et al., 2006; Peyrache et al., 2009; Liu et al., 2012; Ramirez et al., 2013). Furthermore, sensory or electrical stimulation able to synchronize neuronal firing in the millisecond scale has been shown to generate sequentially, in the minute to hour scale, synaptic potentiation, immediate-early gene expression, synaptic remodeling and dendritic sprouting (Chang et al., 1991; Bliss and Collingridge, 1993; Deisseroth et al., 1995; Klintsova and Greenough, 1999). In principle, this sequence of events satisfactorily explains why neurons that fire together wire together, and vice-versa. However, to date there is still a mechanistic hiatus between neuronal synchronization and the perception of complex stimuli, or the planning and execution of complex motor tasks.

The gap between cell assemblies and behavior was anticipated by Hebb (1949), who proposed that synchronized cell assemblies would evolve over time as *phase sequences*: “Any frequently repeated, particular stimulation will lead to the slow development of a ‘cell-assembly,’ a diffuse structure comprising cells in the cortex and diencephalon (and also, perhaps, in the basal ganglia of the cerebrum), capable of acting briefly as a closed system, delivering facilitation to other such systems and usually having a specific motor facilitation. A series of such events constitutes a ‘phase sequence’—the thought process. Each assembly action may be aroused by a preceding assembly, by a sensory event, or—normally—by both.”

For many years these ideas remained untestable, but in the past two decades, the detection and tracking of assemblies became feasible due to major improvements in multi-electrode recording techniques (Nicolelis et al., 2003; Buzsáki, 2004; Schrader et al., 2008), as well as the development of adequate mathematical frameworks for the identification of non-random synchronization (Berger et al., 2010; Denker et al., 2010; Peyrache et al., 2010; Lopes-dos-Santos et al., 2011, 2013). As a consequence, studies on assembly activity and learning were recently published (Peyrache et al., 2009; Benchenane et al., 2010); there were also demonstrations of information coding by the temporal sequence of neurons (Ikegaya et al., 2004; Ji and Wilson, 2006; Pastalkova et al., 2008; Peyrache et al., 2009; Dragoi and Tonegawa, 2010). The hippocampus, in particular, harbors assemblies activated by specific places or time intervals, forming representational sequences (Lee and Wilson, 2002; Macdonald et al., 2011; Kraus et al., 2013; Pfeiffer and Foster, 2013).

In the present work we aimed to advance the investigation of the next logical step in Hebbian theory, namely the detection of phase sequences as consecutive multi-assembly activation patterns. We also set out to investigate the relationship between phase sequences and cognitive behavior. The developed method was based on graph theory and it was applied to datasets comprising chronic extracellular spike recordings from the primary visual (V1) and somatosensory (S1) cortices, as well as the CA1 region of the hippocampus (HP), of rats subjected to a novel object exploration paradigm (Ribeiro et al., 2007).

## Materials and methods

### Experimental periods of the behavioral paradigm

**Figure 1:**
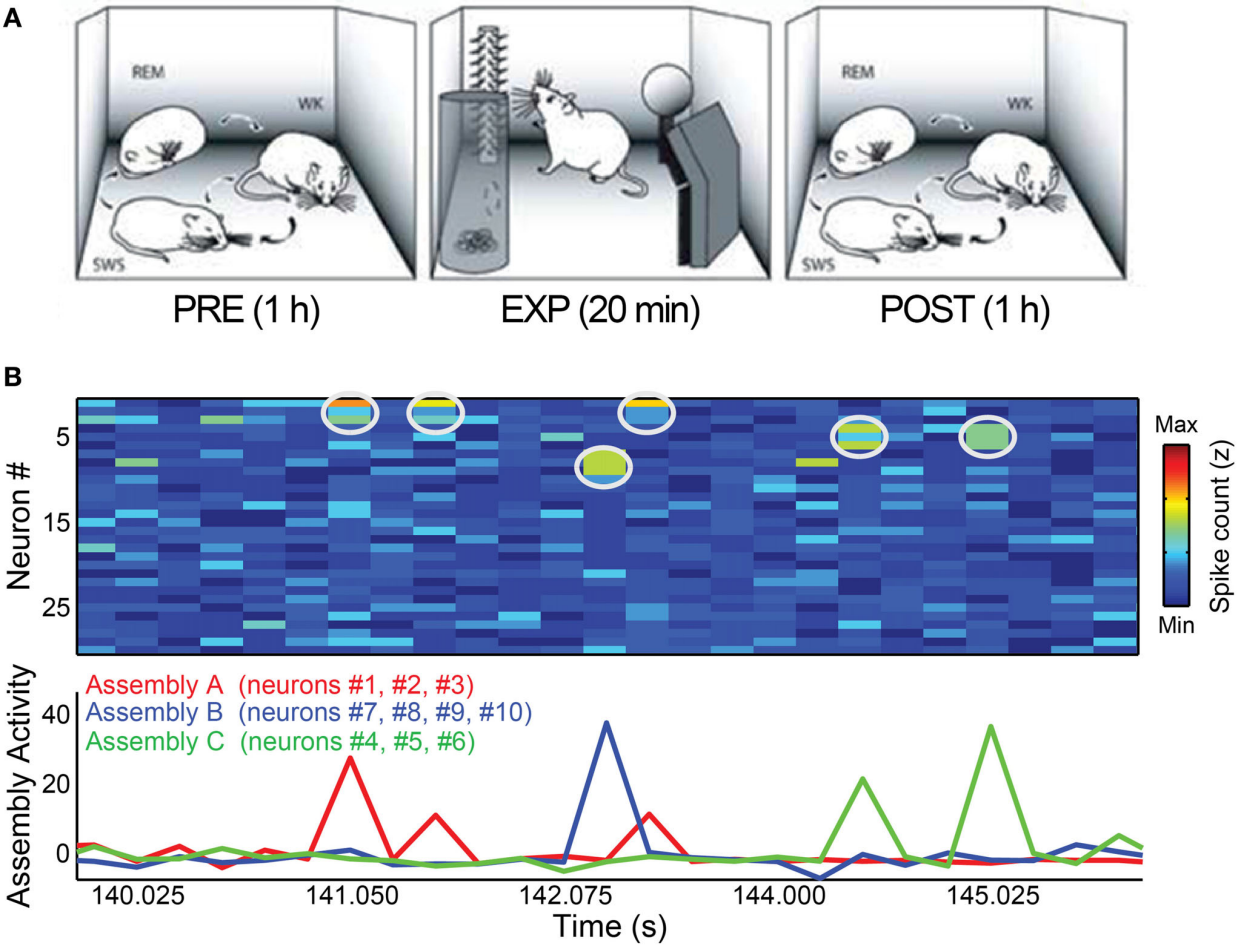
**Behavioral paradigm and cell assembly detection. (A)** Rats were submitted to three periods of experimentation. During PRE and POST periods, animals were kept in a rectangular box freely behaving for 1 h, including complete wake-sleep cycle, sorted here as WK, SWS, and REM. Within EXP period, 4 novel objects were placed in the corners of the box and the animals were free to explore them for 20 min. Figure adapted from Ribeiro et al., (2007). **(B)** Toy example of assembly detection and projection of assembly activity time-series. We simulated 30 independent neurons as Poisson processes with mean 1 spike/bin and created three assemblies (A–C) by setting 3% of the data (1% for each assembly) as bins with synchronization between the cells of a specific assembly. In this dataset, assembly A comprises neurons # 1, # 2, and # 3; assembly B is formed by neurons # 7, # 8, # 9, and # 10 and neurons # 4, # 5, and # 6 make assembly C. Top panel shows the spike matrix (white circles mark co-activations of assembly neurons). Bottom panel shows the assembly activity time-series, calculated using the ICA-based method described in Lopes-dos-Santos et al. (2013). Note that the assembly activities peak only when their corresponding neurons co-fire.

We used data from five Long-Evans adult male rats (300–350 g) recorded before, during and after a novel object exploration paradigm (Ribeiro et al., 2007). The behavioral paradigm began with 1–2 h of recordings as a freely-behaving rat went through the wake-sleep cycle (PRE period). Next, the animal was allowed to explore 4 novel objects placed in the corners of the recording box for 20 min (EXP period). Finally, the objects were removed and the animal was recorded for an additional 1–4 h, freely traversing the wake-sleep cycle (POST period). Video recordings with infrared illumination were used to document behavior. The present study focused on the 1 h PRE and POST periods flanking EXP (Figure 1A).

### Multielectrode array implantation

Briefly, the rats were anesthetized and surgically implanted with multielectrode arrays of tungsten microwires (35 μm, 1.0–1.2 MOhm at 1 kHz). A screw implanted on the frontal portion of the skull served as recording ground. The arrays targeted HP, S1, and V1 in the left hemisphere stereotaxic coordinates in mm from Bregma with respect to the antero-posterior (AP), medio-lateral (ML), and dorso-ventral (DV) axes (Paxinos and Watson, 1997): HP (AP: −2.80; ML: +1.5; DV: −3.30); S1 (AP: −3.00; ML: +5.5; DV: −1.40); V1 (AP: −7.30; ML: +4.00; DV: −1.30). DV measurements were taken with respect to the pial surface. Arrays comprised 16–32 microwires spaced at 250 mm intervals (4 × 4 arrays for S1 and V1, 2 × 16 array for HP). In S1 and V1, arrays were aimed at pyramidal layer V.

### Electrophysiological recordings and unit sorting

As described in detail in Ribeiro et al. (2007), action potentials (spikes) and local field potentials (LFP) were recorded with multi-electrode arrays placed in the dorsal CA1 region and dentate gyrus of HP, in the barrel field of S1, and in V1. Animals were recorded after a 1-week recovery period following surgery. A 96-channel multineuron acquisition processor (MAP, Plexon Inc, Dallas, TX) was used for digital spike waveform discrimination and storage. Action potentials (spikes) were extracted from the high frequency band data and sorted into units using supervised online spike sorting (SortClient 2002, Plexon Inc.) associated with posterior offline validation (Offline Sorter 2.3, Plexon Inc). LFPs recorded from the same wires were pre-amplified, filtered, and digitized using a Digital Acquisition card (National Instruments, Austin, TX) and a MAP (Plexon Inc). Behaviors were recorded throughout the entire experiment under infrared illumination, by way of two CCD video cameras and a videocassette recorder. Video and neural recordings were synchronized with a millisecond-precision timer (model VTG-55; For-A, Tokyo, Japan). Within each region, the amount of units consisted of 42 HP, 33 S1 and 20 V1 for rat # 1, 59 HP, 23 S1 and 28 V1 for rat # 2, 34 HP, 25 S1 and 23 V1 for rat # 3, 39 HP, 27 S1 and 37 V1 for rat # 4 and 45 HP, 39 S1 and 42 V1 for rat # 5.

### Sorting of behavioral states

We used LFP data associated with a behavioral state sorting algorithm (Gervasoni et al., 2004) to classify the states with 1 s resolution. The algorithm is based on a two-dimensional state space defined by two spectral amplitude ratios calculated by dividing integrated spectral amplitudes at selected frequency bands. A scatter plot of the two chosen LFP spectral amplitude ratios (state-space) reveals distinct clusters that correspond to the three major wake-sleep states studied here: waking (WK), slow wave sleep (SWS), and rapid eye movement sleep (REM).

### Assembly detection

A cell assembly is a subset of cells that somehow behave as a single entity. Here we assumed a linear model. More specifically, we defined the activity of a cell assembly as a weighted sum of the activity of individual units. In order to determine the weights of each neuron to each cell assembly we used a recently developed framework (Lopes-dos-Santos et al., 2013), which can be briefly described in four main steps:

1. The spike train of each neuron was binned into 5 ms windows and z-scored (i.e., variance and mean were set to 1 and 0, respectively). Thus, the population activity was transformed in a matrix in which each element represented the normalized number of spikes of a given neuron in a given time bin. We referred to this matrix as *activity matrix*.
2. Then, the number of statistically significant cell assemblies was estimated by counting how many principal components of the activity matrix had associated variances above the upper bound of the Marčenko-Pastur analytical distribution of eigenvalues (Marčenko and Pastur, 1967; Peyrache et al., 2010; Lopes-dos-Santos et al., 2011).
3. The activity matrix was projected into the subspace spanned by the principal components with eigenvalues crossing the statistical threshold and then submitted to Independent Component Analysis (ICA) (Laubach et al., 1999; Hyvärinen and Oja, 2000). Independent components can be understood as *assembly patterns* that represent assemblies when the linear model is assumed (Lopes-dos-Santos et al., 2013), i.e., the values attributed to each neuron in a pattern define the weights of the cells in the corresponding assembly.
4. Individual cell assembly activity was computed by projecting the activity matrix onto its assembly pattern (Lopes-dos-Santos et al., 2013), which can be mathematically defined as:
  $$AA_{b}=\sum_{i\;=\;1}^{N_{neurons}}w_{i}z_{ib}=W^{T}Z_{b},$$
  where *AA*_*b*_ is the assembly activity at time bin *b*, *N*_neurons_ is the total number of neurons, *w*_*i*_ is the weight of neuron *i* in a specific assembly and *z*_*ib*_ is the z-scored activity of neuron *i* within bin *b*. We removed the contribution of single units firing alone (for instance, if a heavily-weighted neuron activated but others were silent, the assembly activity remained low).

Figure 1B shows an illustrative example of an activity matrix (top panel) along with the assembly activities estimated by the method. For more details, see (Lopes-dos-Santos et al., 2013).

## Results

### Time bin determination

**Figure 2:**
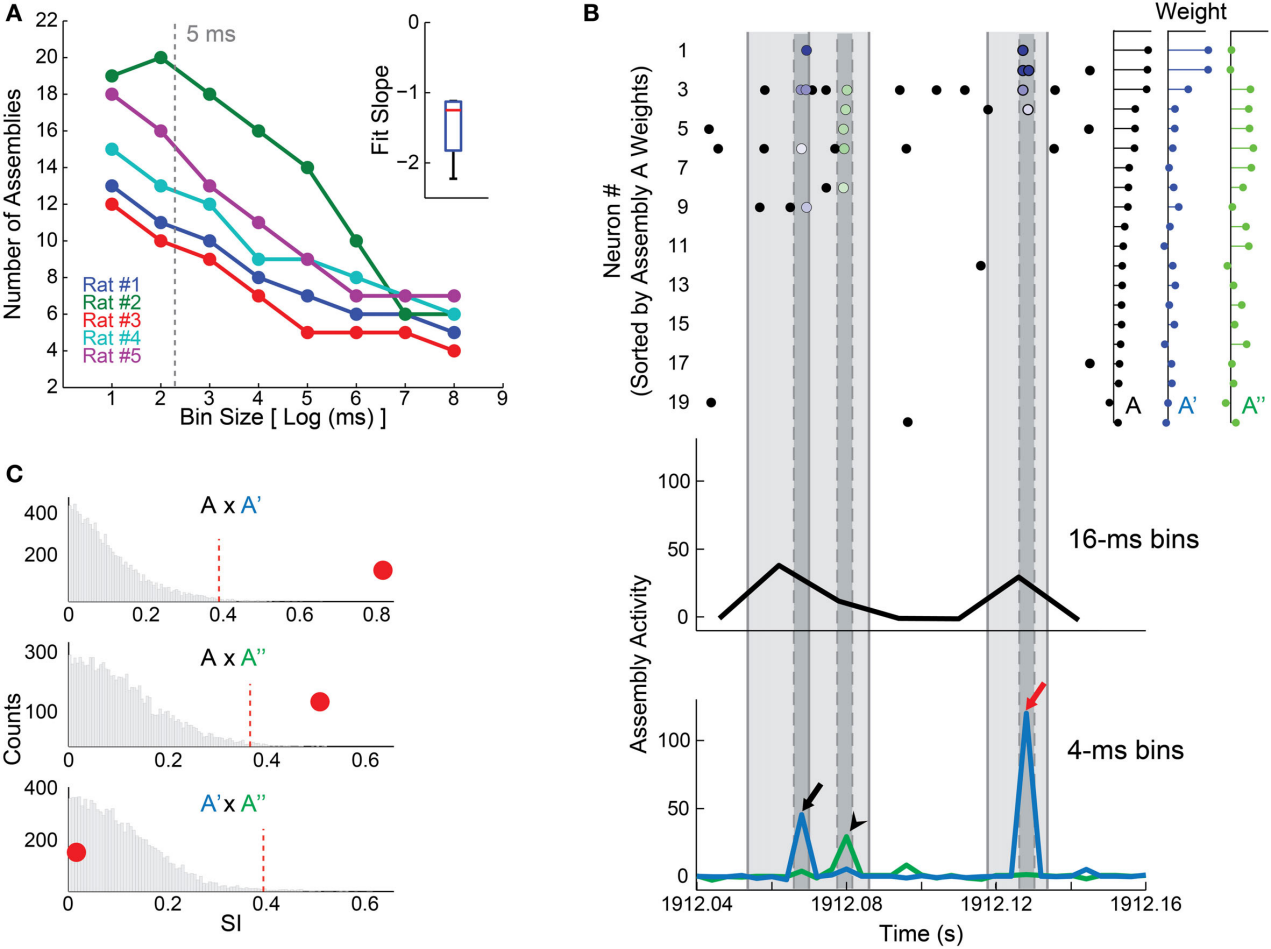
**Time bin size influences the detection of cell assemblies. (A)** Plot between log2 of bin size in milliseconds and the number of detected assemblies. We assessed bins in a binary scale from 2 to 256 ms. Notice an inverse correlation between log2 of bin size and the number of assemblies; inset shows the distribution of slopes of the linear fits in the main panel. Gray dashed line depicts the 5 ms bin size chosen in our study. **(B)** (Bottom) 120 ms of assembly activity from animal # 1, showing activity of assembly A (black line), detected in EXP WK with 16 ms bin size, and of assemblies A′ (blue line) and A″ (green line), detected with a bin size of 4 ms. (Top) Rasterplot of the 20 most relevant neurons that constitute assembly A (80% of the weight), ranked from highest weight to the twentieth highest. Light gray shadow represents 16 ms intervals, dark gray ones represent 4 ms. Blue dots exhibit the spike times of neurons contributing to assembly A′ activity peak (black and red arrows, bottom panel). Green dots mark spikes contributing to assembly A″ activity peak (black arrow head, bottom panel). Colored dots (spike times) are graded from darker to lighter respective to the weight of the correspondent neuron in the assembly pattern. Note that neurons participating in assembly A (bin 16 ms) were sorted into assemblies A′ and A″ (bin 4 ms), which can be active in sequence (black arrow and arrow head) or independently (red arrow). **(C)** Exploring similarities between assemblies. Panels show the histogram of SI values from 10,000 comparisons made by shuffling the neurons weights within assemblies to build a null hypothesis (bootstrap procedure). Red dashed line shows the threshold for significance at *p* = 0.01. Red circles depict the SI between A and A′ (0.82, top), A and A″ (0.51, middle), and A′ and A″ (0.016, bottom). Note that assembly A is significantly similar to A′ and A″ (*SI* = 0.82 and 0.51, respectively). The SI between A′ and A″ was small (*SI* = 0.016), indicating that, in addition to the fact that these assemblies have independent activity, they also have orthogonal membership. A′ and A″ exhibit strong assembly activations at different time bins (panel **B**–arrows vs. arrow head). However, when 16 ms time bins were used, the activities of these assemblies were packed in the same time window, causing the merge of A′ and A″ into A.

We used an empirical approach to adequately choose the size of the time bins. First, we tested a wide range of bin sizes (2–256 ms) to investigate the relationship between bin size and number of detected assemblies. As shown in Figure 2A, we found an inverse relation between bin size and number of assemblies. We analyzed this closely and found that single assemblies detected with larger bin sizes could be split in two other assemblies when smaller bin sizes were used. The raster plot in Figure 2B shows the 20 most weighted units, sorted from heavier (top) to lighter (bottom), which comprise the patterns of assembly A (80% of the total weight). This assembly is one of the assemblies detected using a 16 ms time bin in rat # 1 dataset, and its activity is shown in black (Figure 2B, bottom); while the activities of two assemblies detected using a 4 ms bin size (A′ and A″) are depicted in blue and green, respectively (Figure 2B, bottom).

To use a quantitative criterion to compare assembly composition, a Similarity Index (SI) was defined as the absolute value of the inner product between the assembly patterns (unitary vectors) of two given assemblies, varying from 0 to 1. Thus, if two assemblies attribute large weights to the same neurons, SI will be large; if assemblies are orthogonal, SI will be zero. We applied a permutation test in order to determine whether SIs were significantly above chance. This test consisted in shuffling the weights of each pattern across neurons, and then recalculating the SI. We ran 10,000 permutations in order to construct a null hypothesis distribution. Two patterns were regarded as *representations* of the same assembly if their original SI was larger than the 99th percentile of the null hypothesis distribution (i.e., *p* = 0.01). Using this process, we found that both A′ and A″ were significantly similar to assembly A (Figure 2C). This indicates that units with larger weights in assembly A were split in two independent (*SI* = 0.016) assemblies A′ and A″ comprising partially non-overlapping sets of units (respective action potentials indicated by blue and green dots in the raster plot of Figure 2B, respectively). Considering that large bin sizes may conceal fast assembly sequences (Figure 2B), we chose the 5 ms bin as a compromise between a high temporal resolution and the need to avoid small bin sizes close to the neuronal refractory period.

### Searching for assemblies in different experimental periods

After defining bin size, we focused on the assessment of the differences among assemblies detected using spike matrices from different experimental periods (PRE, EXP and POST). Our goal was to investigate whether the exposure to novel objects changes the assembly repertoire. At first we ignored sleep states and extracted assembly patterns from entire PRE, EXP and POST-WK periods (each one independently). Next, we used the SI to compare all assemblies between experimental periods.

**Figure 3:**
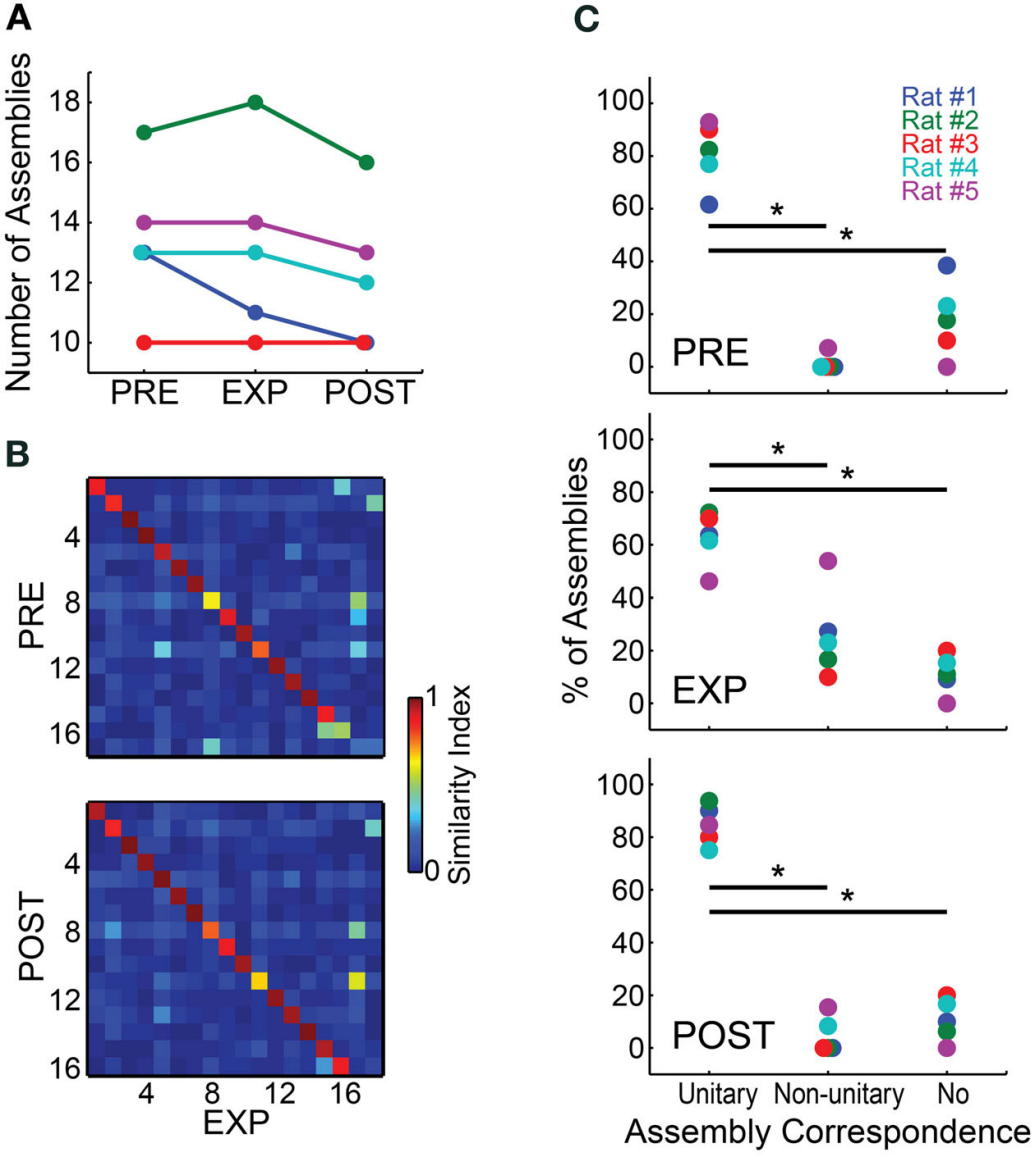
**Cell assemblies are highly conserved across experimental periods. (A)** Number of assemblies detected using spike matrices from the different experimental periods. **(B)** SI values among assembly patterns of rat #2 across experimental periods. Assembly patterns were detected using a 5 ms bin size. Assembly labels were sorted to let highest values in the main diagonal. **(C)** For each experimental period, the panels show the percentages of assemblies within each of the categories defined by the number of significant correspondences between the assemblies of a given experimental period and the assemblies from flanking periods (from top to bottom, PRE, EXP and POST). Two assembly patterns were deemed correspondent if their SI was above a threshold set by a bootstrap procedure (*p* = 0.0001). The categories were defined as unitary correspondence, non-unitary correspondence and no correspondence, representing the percentage of assemblies within rats that showed, respectively, a single correspondence between flanking periods, two or more flanking correspondences, or no correspondence whatsoever. Note that the percentage of assemblies within the unitary correspondence category was considered significantly higher than the other categories for all experimental periods (Wilcoxon ranksum test, ^*^p < 0.05, Bonferroni corrected).

We found little variation in the numbers of assemblies across different experimental periods (Figure 3A). Most animals showed a maintenance or minor decrease in the number of assemblies from PRE to EXP, except for rat # 2, which showed an increase of one assembly. From EXP to POST, the number of assemblies detected also dropped slightly, except for rat # 3, which showed a stable number of 10 assemblies per period. Rat # 1 showed the highest variance in the number of assemblies detected across periods, ranging from 13 in PRE to 10 in POST. Figure 3B illustrates the substantial similarity between assemblies detected in different experimental periods for rat # 2, which overall showed the largest number of assemblies. To assess assembly conservation across experimental periods, we then categorized the assemblies within each experimental period as showing unitary correspondence, non-unitary correspondence, or no correspondence. An assembly was considered to show unitary correspondence when it was significantly similar to only one assembly in each of its flanking experimental period(s) with *p* < 0.0001; non-unitary correspondence defined assemblies which showed more than one correspondence or, in the case of EXP, those with correspondence to one assembly from a flanking period but not with the other (e.g., correspondence with PRE but not with POST); the no-correspondence category comprised assemblies showing no significant correspondences. Group results across different experimental periods (Figure 3C) show that the number of assemblies exhibiting unitary correspondence was significantly higher than those showing non-unitary correspondence or no correspondence, including EXP which is flanked by two neighbor periods (Wilcoxon ranksum test, *p* < 0.05, Bonferroni corrected).

A comparison across experimental periods reveals that the percentage in PRE of assemblies with no correspondence was slightly elevated, while non-unitary correspondence was very minor. During EXP the percentage of non-unitary correspondences increased, while the percentage of unitary correspondences and no-correspondences decreased. This could represent the fact that EXP is flanked by two neighbor periods, while PRE and POST are flanked by only one. Another possible explanation is that the exposure to novel objects could have changed some assembly activation patterns, increasing their co-activations (see Figure 6B), and causing separate assemblies to be detected as one. This may decrease the SI, leading to non-significance between similar assemblies, and/or to significant similarity of one assembly with two or more assemblies from flanking periods, comprising significant but lower SIs. The POST period showed the highest percentages of assemblies in the unitary correspondence category, with a very small percentage of assemblies in the non-unitary and no-correspondence categories. This indicates that the typically smaller number of assemblies in POST (Figure 3A) comprises a subset of assemblies that is essentially the same as in EXP. Across all animals, we found an average of only one EXP assembly per rat that showed no correspondence to any PRE assembly, and yet had correspondence with a POST assembly. This points to a very high conservation of assemblies across experimental periods, and rules out the possibility that new assemblies are formed within EXP and reverberate during POST. For this reason, we continued our investigation of assembly sequences by extracting the assembly patterns from a concatenated spike matrix of all WK intervals (PRE+EXP+POST), and then projecting the assembly activity over the entire recording, throughout the wake-sleep cycle. Using this approach, we detected 11, 18, 10, 13 and 13 assemblies for rats # 1 to # 5, respectively.

### Detecting assembly activations

**Figure 4:**
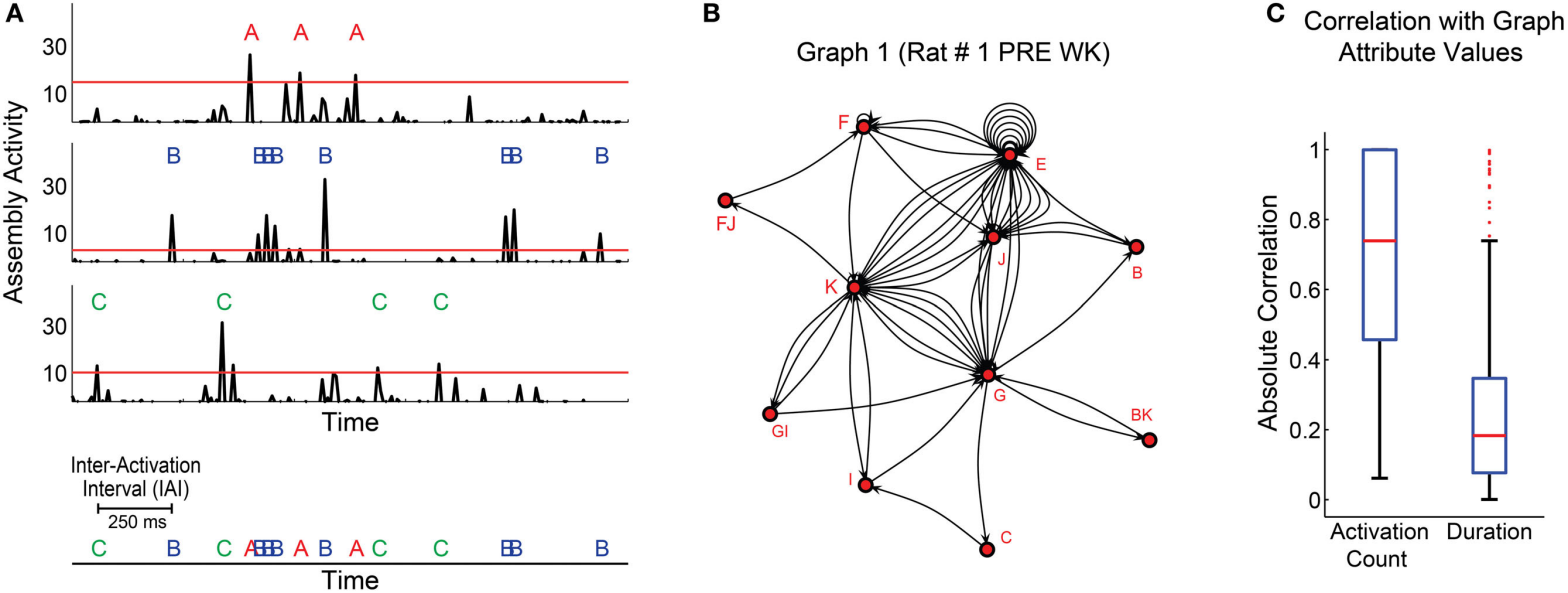
**Determination of sequences of cell assembly activations**. **(A)** 1.5 s interval showing activity of 3 assemblies (A–C) of rat # 1 (3 top panels). Thresholds are the 99th percentiles of the activity values for each assembly. Threshold-crossing peaks are considered assembly activations. Assembly activation sequence is defined as the series of activation across different assemblies within subjects; and the time interval between two subsequent activations is called inter-activation interval (IAI) (bottom panel). **(B)** Exemplary graph generated with assembly activations from the first WK episode of rat # 1 during PRE. **(C)** Distribution of absolute Pearson correlation values between graph attributes and two other variables: activation count and graph duration. Graphs were generated using assembly activation sequences from behavioral states' episodes. Panel shows distribution of data from all episodes. Note that activation count was generally correlated with graph attribute values in our dataset (median = 0.74, 74% of correlations were significant with *p* < 0.05), while the graphs duration were not (median = 0.18, 8% of correlations were significant with *p* < 0.05).

In order to improve the time resolution for the analysis of assembly activation sequences, we first re-binned the spike trains from each unit using 1 ms bins, and convolved the data with a Gaussian kernel (maximum = 1, 80% of the AUC within 5 ms windows). Then we projected the activity of all assemblies, and defined a threshold (for each assembly) as the 99th percentile of the distribution of activity values across time bins (Figure 4A, red lines). Figure 4A shows the activity of three exemplary assemblies (A, B, and C) from rat # 1, which above-threshold peaks are depicted by red, blue and green letters (assembly activations), respectively. Subsequent assembly activation was only considered after a “refractory” period of 3 ms elapsed.

### Calculation of assembly graph attributes

We constructed the assembly activation sequence by labeling and concatenating assembly activations from different assemblies (Figure 4A, bottom). Graphs were built from this sequence, so that each assembly corresponded to a node, each edge corresponded to the temporal sequence of consecutive node activations, and the time intervals between two assembly activations were considered inter-activation intervals (IAI) (Figure 4A, bottom). The coactivation of two or more assemblies within the same time bin was represented as an additional node in the graph, whose label comprised the labels of the assemblies activated at the same time. For instance, if assemblies F and J displayed synchronous activation, a fourth node FJ was added to the graph, always in the alphabetical order (Figure 4B).

Two parameters shaped the graphs: maximum IAI and number of activations per graph (activation count). The maximum IAI parameter defined the threshold IAI within each graph, i.e., every time interval between assembly activations within a graph should be less than or equal to this maximum IAI. Seven different maximum IAI values ranging from 10 to 1000 ms were explored.

An initial assessment of the data varying only the maximum IAI criterion showed that, in general, the assembly graph attributes were proportional to the activation count in a graph (Figure 4C, median of absolute Pearson correlation indexes distribution = 0.74), while the duration (the interval between the first and last assembly activation within a graph) was not correlated to assembly graph attributes (Figure 4C, median of absolute Pearson correlation indexes distribution = 0.18).

A fixed number of assembly activations per graph was used to control for this variability in the graph attributes. Since the minimum activation count necessary to maximize the density of a graph (Table 1) is the square of the number of nodes –*Number of Assemblies*^2^, we evaluated seven values of activation count as percentages of *Number of Assemblies*^2^ (10, 20, 50, 100, 120, 150 and 200%). The custom-made java software *Speechgraphs* (Mota et al., 2012; http://neuro.ufrn.br/softwares/speechgraphs) was used to calculate 13 assembly graph attributes (Table 1).

**Table 1:** **Graph attributes**.

**Abbreviation**	**Name**	**Definition**
Nodes	Number of Nodes	Number of assemblies activated and single sets of co-activations in the graph
RE	Repeated Edges	Number of edges linking the same pair of nodes more than once in one specific direction
PE	Parallel Edges	Number of edges linking the same pair of nodes more than once irrespective of the direction
L1	Loops with one node/Self-Loops	Number of edges between one node and itself
L2	Loops with two nodes	Number of pairs of edges between two nodes one in each direction
L3	Loops with three nodes	Number of sets of three edges in one specific direction leaving one source node, passing through two other nodes and coming back to the source node
LCC	Largest Connected Component	Number of nodes comprising the largest sub-graph in which each node is connected to each other through a path in the sub-graph (applied to the undirected version of the graph)
LSC	Largest Strongly Connected Component	Number of nodes comprising the largest sub-graph in which all nodes are mutually reachable, i.e., there is a path from node A to node B, and one from node B to node A (applied to the directed version of the graph)
ATD	Average Total Degree	Mean of the number of edges pointing to or departing from a node, across nodes
Density	Density of the graph	Density number that goes from 0 to 1 representing the percentage of possible edges that really exist in the graph
Diameter	Diameter of the Graph	Length of the longest shortest path between the node pairs of a network
ASP	Average Shortest Path	Average length of the shortest path between pairs of nodes of a network
CC	Clustering Coefficient	Average across nodes, of the percentage of real edges between the neighbor nodes of a node over the total possible edges between these neighbors

### Changes in population rate do not explain the activity of individual assemblies

The algorithm to algebraically define assembly activity was the squared linear combination of the firing rate of the units in a given time bin (Lopes-dos-Santos et al., 2011, 2013). Hence, while assembly activity is dependent on population firing rate, it is not fully determined by it, because its projection also depends on the weight of each unit on that specific assembly.

**Figure 5:**
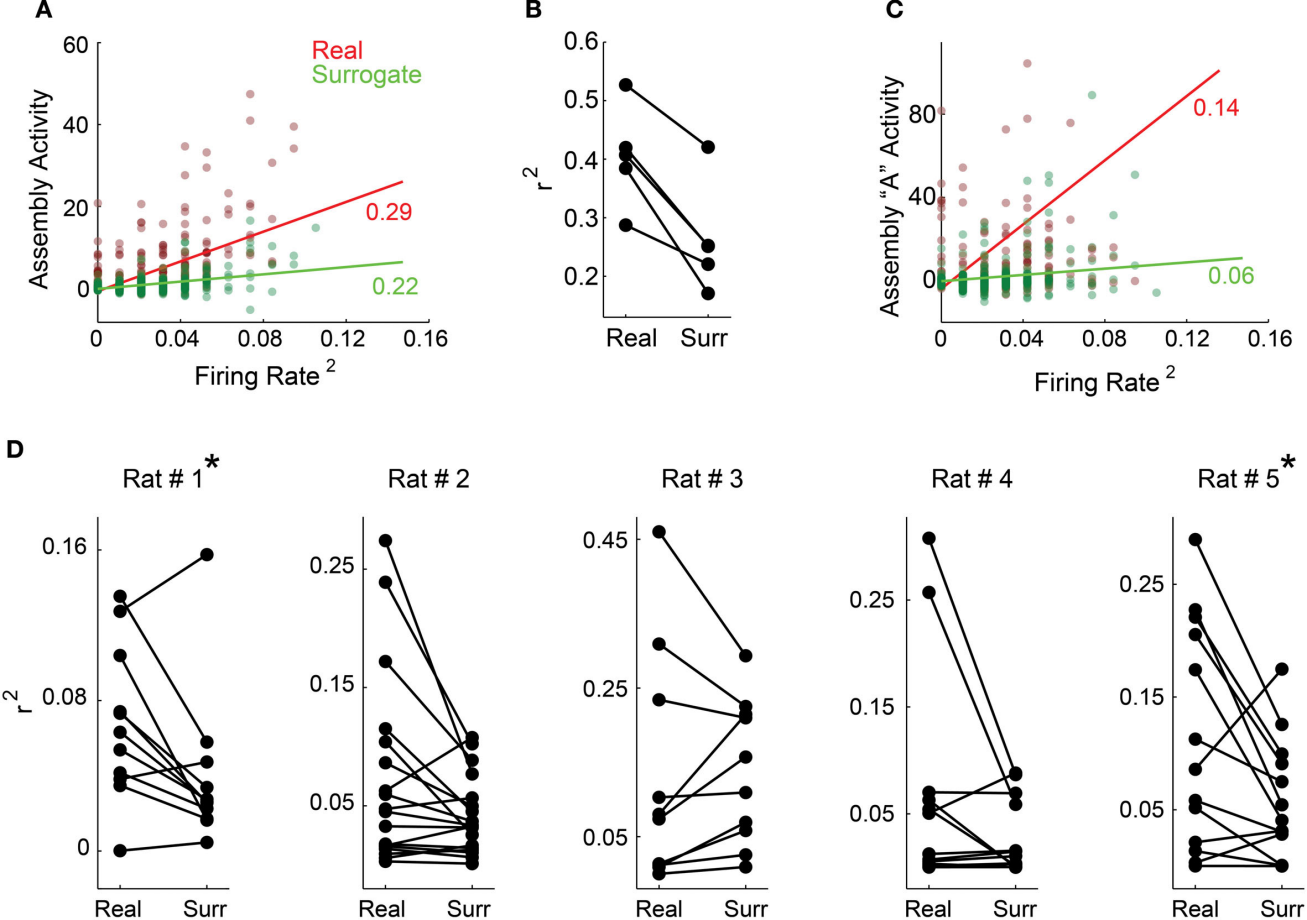
**The activity of individual assemblies is not reducible to rate fluctuations. (A,C)** show exemplary panels from rat # 1 and **(B,D)** show group data. **(A)** Squared mean of the population rate and the mean of all assemblies' activity within each 1 ms bin (dark red dots). In order to scramble associative behavior and keep the firing rate fixed, we also plotted the mean population rate against the mean assemblies' activity projected using the spike matrix with neurons' labels surrogated within each time bin (dark green dots). Light red and green lines depict the least square linear fit for each color coded subset of points along with the correspondent coefficients of determination (*R*^2^). **(B)** Coefficient of determination distribution for all rats. For all animals, data surrogation impaired the correlation between firing rate and assembly activity. **(C)** The same color code as in **(A)**, but plotting the mean population rate against the activity of a single exemplary assembly from rat # 1. **(D)** Shown are distributions of all rats *R*^2^ values for the linear fits from the correlation between mean population rate and individual assemblies' activity (left) and mean population rate and individual assemblies' activity estimated from surrogated spike matrices (right). Note that both distributions exhibit very low *R*^2^ values and that there is a decreasing trend from real to surrogated data, with significant difference for rats # 1 and # 5 (^*^p < 0.05, Wilcoxon signed-rank paired test).

A plethora of studies have shown that firing rate changes convey behavioral information (Adrian and Zotterman, 1926; Hubel and Wiesel, 1959; O'Keefe and Dostrovsky, 1971; Moritz et al., 2008); thus, it was first important to show that assembly activity is not just an epiphenomenon of population rate. To address this issue, we plotted the squared mean population rate against the mean of all assemblies' activity within each bin along the whole experiment for each rat (Figure 5A for rat # 1, dark red dots). The *R*^2^ of the linear fit between these two variables was low for all animals (Figure 5B), indicating that they display a weak correlation. We then plotted the same squared mean of the population rate against the mean assembly activity projected using spike matrices with surrogated rates within each single bin (Figure 5A, dark green dots). This allowed us to vary one of the variables that define assembly activity (weights of each unit within each assembly), while keeping the other unchanged (population rate). This approach showed linear fits with even lower *R*^2^ values (Figure 5A, light green line for rat # 1 and Figure 5B for all rats).

Next we investigated activity time-series of individual assemblies (Figure 5C, exemplary assembly from rat # 1). Figure 5D shows *R*^2^ values for the linear fits from all individual assemblies as in Figure 5C, for all animals (real data—left; surrogated data—right). All values are very low, and become even lower when we use the surrogated dataset, including a statistically significant difference in *R*^2^ values between real and surrogated datasets, for rats # 1 and # 5. (Figure 5D, asterisk, Wilcoxon signed-rank paired test, *p* < 0.05). Altogether, these results indicate that the activity of individual assemblies is not reducible to fluctuations of the population firing rate.

### Assembly activation rate and coactivations

**Figure 6:**
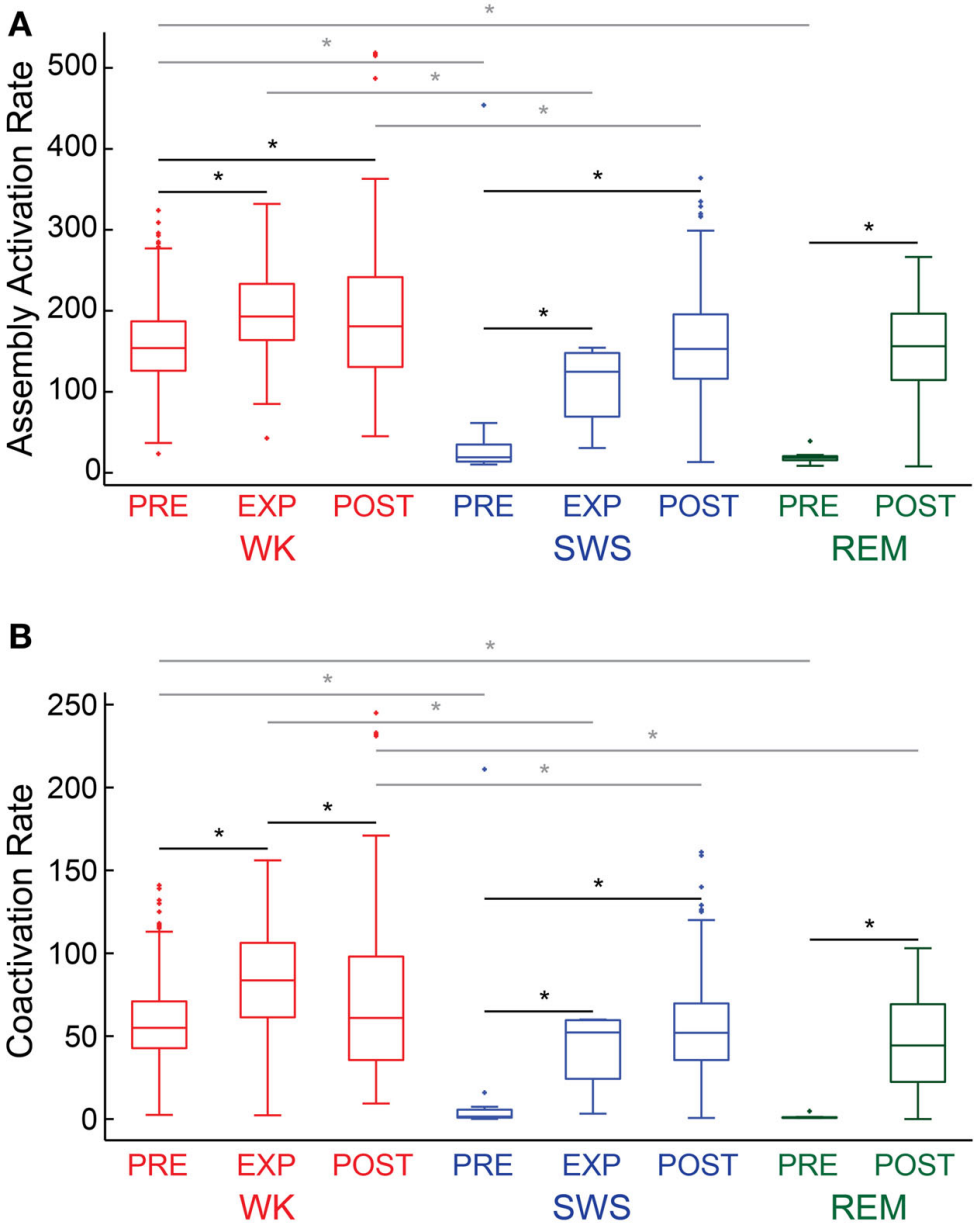
**Descriptive statistics**. Panels show the distribution of assembly activation rate **(A)** and co-activation rate **(B)** (events per second) during different behavioral states and experimental periods for rat # 5. Behavioral states boxplots are color coded as red, blue, and green for WK, SWS and REM, respectively. Experimental periods (PRE, EXP and POST) are placed together and in chronological sequence within each behavioral state. Black lines with asterisks reflect significance between two different experimental periods within a given behavioral state. Gray lines with asterisks reflect significance between two different behavioral states within a given experimental period (*p* < 0.05, bootstrap corrected for multiple comparisons).

We analyzed assembly activation time-series (Figure 6A, exemplary plot from rat # 5) from all behavioral states (WK, SWS and REM) and experimental periods (PRE, EXP and POST). Considering all rats, we found that the assembly activation rate during WK was significantly higher in almost all the paired comparisons (18 out of 21) of experimental periods between behavioral states (gray lines with asterisk, *p* < 0.05, Wilcoxon ranksum test, bootstrap corrected). Moreover, in all rats the assembly activation rate during POST SWS was significantly higher than during PRE SWS (Figure 6A, exemplary plot from rat # 5, black line with asterisk), which suggests that the increase in firing rates after novel object exploration (Ribeiro et al., 2007) may underlie the elevated co-firing of assembly neurons. Interestingly, two out of the three rats that displayed REM during PRE and POST, showed elevated activation rate after the experience. Previous work with larger groups including the present dataset showed no significant firing rate change between PRE REM and POST REM (Ribeiro et al., 2007). The distribution of assembly coactivations followed the same pattern of the assembly activation rate, in which POST SWS displayed higher values than PRE SWS for all rats. The number of coactivations was also higher during WK than during sleep (Figure 6B, exemplary plot from rat # 5); with significant differences in 18 out of 21 possible comparisons.

### Graph analysis

**Figure 7:**
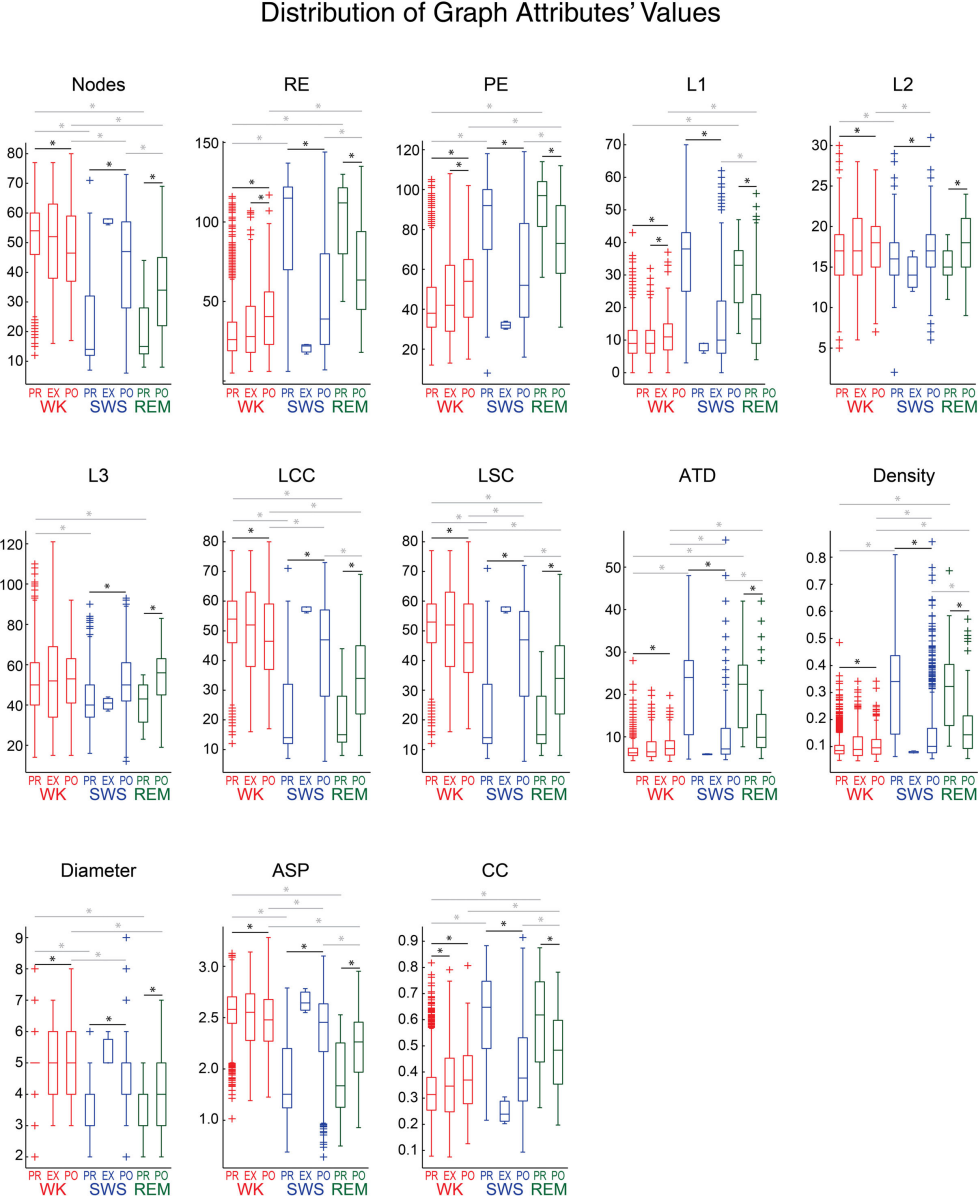
**Assembly graph attributes vary significantly across behavioral states and experimental periods**. Panels show the distribution of graph attributes' values from rat # 5, using 1 s maximum IAI and 169 activations/graph, for different behavioral states and experimental periods. As in Figure 6, behavioral states boxplots are color coded as red, blue, and green for WK, SWS and REM, respectively. Experimental periods (PRE, EXP and POST) are placed together and in chronological sequence within each behavioral state. Black lines with asterisks reflect significance between two different experimental periods within episodes of a given behavioral state (*p* < 0.05, Wilcoxon ranksum test, Bonferroni corrected). Gray lines with asterisks reflect significance between two different behavioral states within a given experimental period. Note that nearly all the attributes sorted WK from SWS, during PRE or POST (except for L1 during PRE and L3 during POST). WK was significantly different from REM during PRE (12 attributes) and POST (11 attributes), SWS was significantly different from REM during POST (10 attributes), but no attribute could sort SWS and REM during PRE. Only one attribute was capable of sorting PRE from EXP within WK. When comparing PRE × POST within WK, 12 attributes could separate them. EXP WK graphs were detected as different from POST WK graphs by 3 attributes. PRE SWS could be sorted from POST SWS, and PRE REM could be sorted from POST REM, using any of the graph attributes studied.

**Figure 8:**
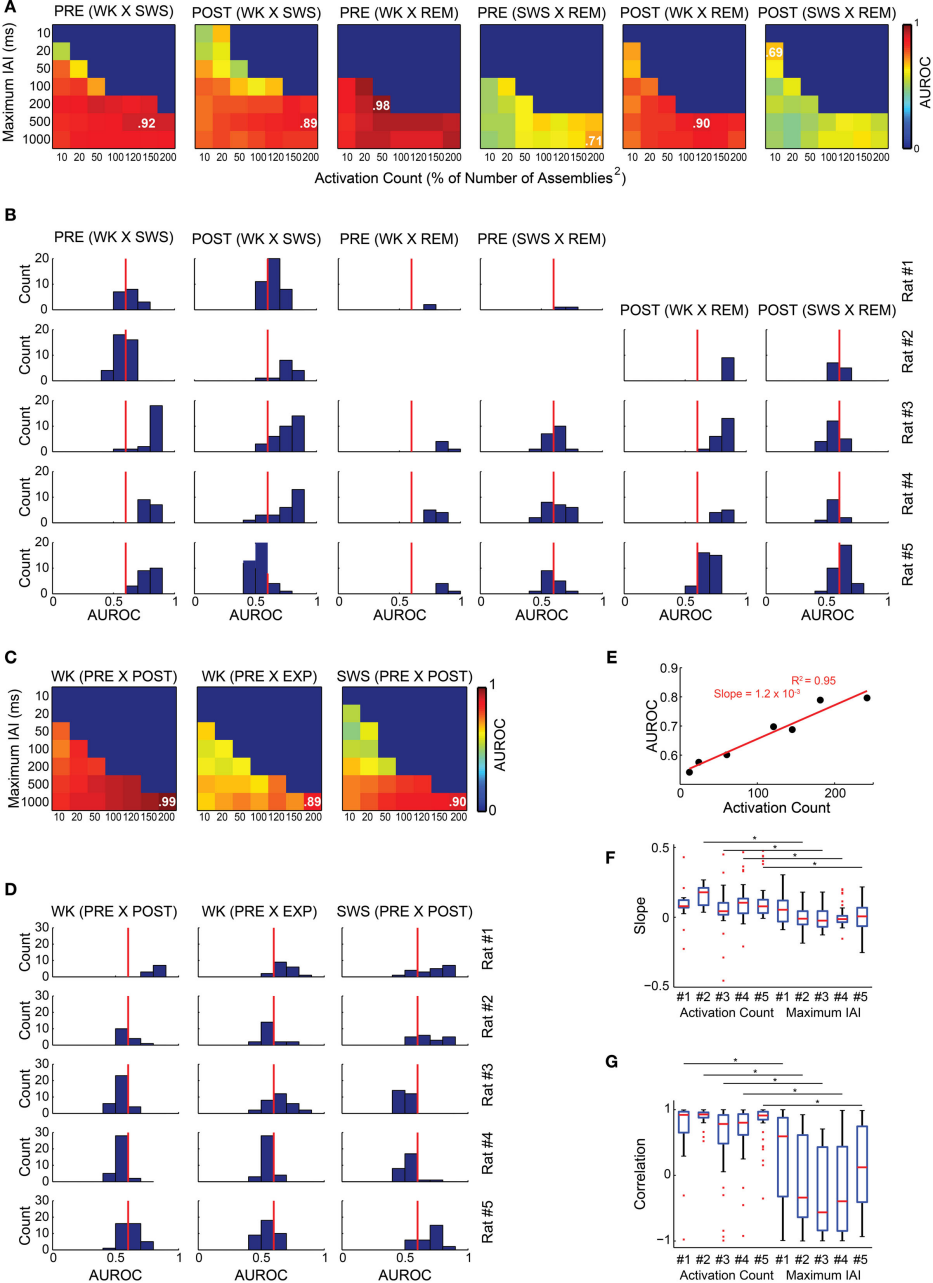
**Assembly graph attributes allow for the automatic classification of experimental periods and behavioral states**. **(A,C)** The rows of each panel represent the graphs maximum IAI (within the graph, every IAI is less than or equal to the maximum IAI value), while the columns correspond to the number of activations within the graphs defined as percentages of the squared number of assemblies. Color codes vary from 0 to 1 and represent the median AUROC of 50 classifications made for 20 random graphs from each of the experimental periods compared using a Naïve Bayes classifier; e.g., 20 graphs from PRE WK compared with 20 graphs from EXP WK. In some cases of the parameter screening, we could not obtain the minimum 20 graphs necessary for the classification. For instance, it was impossible to generate one single graph comprising 200 activations (200% of *Number of Assemblies*^2^ for rat # 3) within the 10 ms maximum IAI. These conditions were coded blue to indicate no classification. The maximum AUROC value of each panel is indicated. **(A,B)** Sorting of behavioral states. **(A)** Panels show the classification quality across different maximum IAI and activation count values for rat # 4. **(B)** Histograms of AUROC values as in panel **(A)** for all rats. Red line depicts the 0.6 AUROC value, which sets the lower bound for a good classification quality. WK and REM were well sorted by graph attributes, with maximum AUROC values ranging from 0.68 to 0.98 for all rats within both PRE and POST periods. The sorting of SWS and REM was substantially less accurate, with maximum AUROC = 0.78 during POST in rat # 5. The sorting between WK and REM was very good for all rats during PRE, (maximum AUROCs from 0.78 to 0.98). **(C,D)** Sorting of experimental periods. **(C)** Panels show the classification quality for rat # 1 across different maximum IAI and activation count values. **(D)** Histograms of AUROC values from the panels as in panel **(C)** for all rats. All the comparisons yielded maximum AUROCs ranging from 0.55 to 0.99. **(E)** Correlation between AUROC and activation count using a 1000 ms maximum IAI from rat # 1 graphs comparing PRE WK and PRE SWS. The slope of the linear fit indicates that each single activation added to a graph, adds 0.0012 to the AUROC, with activation counts varying from 12 to 242 (AUROCs vary from 0.54 to 0.80). **(F)** Distribution of slopes of the linear fits between activation count and AUROC with fixed maximum IAI value (e.g., panel **E**); and between maximum IAIs and AUROC with fixed activation count. We used the AUROCs from all the comparisons and conditions (maximum IAI and activation counts) for all animals and considered only fits with three or more data points. The analysis shows that the maximum IAI contribution to the AUROC is around zero (mean across rats = 0.0024) and even negative, while the contribution of the activation count is divergent, with a clear majority of positive contributions (mean across rats = 0.097), yielding a significant difference between these two variables, except for rat # 1 **(G)** Distribution of Pearson correlations indexes for the comparisons in panel **(F)**. Note that activation count shows strong positive correlation with AUROCs (medians = 0.92, 0.93, 0.78, 0.80, and 0.91 for rats # 1 to # 5, respectively; 53% of the values with *p* < 0.05), while maximum IAIs are scattered, with values spanning the entire scale, and medians closer to zero or even negative for all rats (0.59, −0.33, −0.56, −0.39, and 0.12 for rats # 1 to # 5, respectively; 8% of the values with *p* < 0.05). Asterisks indicate significant differences between activation count and maximum IAI distributions of correlation values within the same animal.

We found that graph attributes varied significantly across behavioral states and experimental periods (Figure 7). We tested therefore whether a Naïve Bayes classifier could extract, from the assembly graph attributes, information enough to sort behavioral states and experimental periods (John and Langley, 1995). We used the java software *Weka* (http://www.cs.waikato.ac.nz/ml/weka/) to perform the classifications and estimated their quality by the area under the receiver operating characteristic curve (AUROC). Figures 8A,B show that it was possible to sort behavioral states with very high quality of classification, particularly when WK and REM were compared (maximum AUROCs ranging from 0.78 to 0.98). WK and SWS could also be distinguished, at a somewhat lower level (maximum AUROCs ranging from 0.69 to 0.96). The poorest quality of classification was obtained by sorting SWS from REM (maximum AUROCs ranging from 0.64 to 0.78).

The classification quality across experimental periods was not as good as across behavioral states (median across rats 0.57 vs. 0.69, Wilcoxon ranksum test, *p* < 0.01), except for rat # 1. Figures 8C,D show that the maximum AUROC values for the comparisons between experimental periods ranged from 0.55 to 0.99, with distribution of all values yielding 0.52 and 0.67 as the first and third quartiles, compared to 0.58 and 0.84, as quartiles for the comparisons between behavioral states. We found a strong positive correlation between the AUROC of graph attributes and activation count for all the comparisons made (e.g., rats # 4 and # 1 in Figures 8A,C). One example of this correlation is shown on a plot of the AUROC values from the classification between PRE WK and PRE SWS vs. the activation count of the graphs of rat # 1, considering only values obtained using the 1000 ms maximum IAI (Figure 8E). The figure shows a positive correlation associated with an extremely strong linear fit (*R*^2^ = 0.95) and a 1.2 × 10^−3^ slope, in association with major variation in AUROC values (full range: 0.54–0.80). To test if this was a general effect of assembly count on AUROCs and to analyze the general effect of maximum IAI on AUROCs, we plotted the AUROCs vs. the activation counts along a constant maximum IAI; and the AUROCs vs. the maximum IAIs considering a constant activation count for the panels from all rats. Note that activation count accounts for AUROC variability significantly more than the maximum IAI, except for rat # 1 (Figure 8F), according to a positive correlation (Figure 8G). It is important to note that there was no AUROC above 0.68 when we used maximum IAIs below 20 ms. Maximum AUROCs were obtained using each of the seven different activation counts explored.

## Discussion

Our results show that assembly graphs comprising synchronized neuronal units recorded from the hippocampus and primary sensory cortices can be used to sort behavioral states (maximum AUROC values ranging from 0.64 to 0.98) and experimental periods (maximum AUROC values ranging from 0.55 to 0.99) before, during and after novel object exploration. This sorting is based on several attributes that reflect the structural properties of assembly graphs. At this point we do not know whether these attributes are informative due to a causal relationship with behavior, or as an epiphenomenon of some other underlying cause. In all, our investigation corroborates the notion that phase sequences, understood as specific patterns of assembly activations, reflect the different regimes of neural processing as animals traverse the wake-sleep cycle and acquire novel information about the environment.

Such interpretation of the results cannot be furthered without addressing the problem of the arbitrary definition of time scale for synchronous firing. As shown in Figure 2A, the number of assemblies detected decreases with bin size. We showed evidence that this may be due to the tight temporal association of assemblies detected using smaller bin sizes, which are detected as a single assembly when larger bin sizes are used. Our choice of bin size = 5 ms for the generation of assembly graphs, well within the potentiation window of spike time dependent plasticity (STDP) (Bi and Poo, 1998), represents a compromise between the number of assemblies detected and the need to avoid extremely low bin sizes near the neuronal refractory period.

Our results show that the repertoire of assemblies is almost unchanged across experimental periods, which suggests that novel experience does not create new assemblies in the hippocampus and primary sensory neocortex of the normal adult rat. Our finding is compatible with Hebb's hypothesis that assemblies correspond to the primitive building blocks of representations, being slowly formed across development but nearly unchanged in adulthood. The experience-dependent changes in the structure of assembly graphs, revealed by the use of a classifier, also corroborates the complementary Hebbian hypothesis that relevant information about concepts, percepts and behavior in general is coded at the level of multiple assembly activations, the so called phase sequences (Hebb, 1949).

We also showed that the activity of single assemblies cannot be reduced to the changes in firing rate. Changes in neuronal firing rates constitute well-known indexes of behavior (Adrian and Zotterman, 1926; Hubel and Wiesel, 1959; O'Keefe and Dostrovsky, 1971; Moritz et al., 2008). If phase sequences are indeed important to generate new neural representations, they should carry more specific information than firing rates. Since assemblies are subsets of neurons that function transiently as closed systems, the neurons related to a given perception or behavior should have their rates affected synchronously, so as to be detected as assemblies. The calculation of assemblies and the projection of their activity is a way to reduce the dimensionality of a population of neuronal units onto neuronal subsets which are likely related to behavior. Investigation of whether phase sequences carry more information than firing rates is ongoing.

The automatic sorting of behavioral states using the attributes of assembly graphs reached a very high level, but the sorting of experimental periods was substantially less accurate. The major behavioral states comprise markedly different physiological patterns in the brain (Noda et al., 1969; Vanderwolf, 1969; Hobson and McCarley, 1971; Gervasoni et al., 2004), likely not the case for the experimental periods investigated here. One possible cause for this difference may be the small amount of assemblies detected, due to the under-sampling of the neuronal units actually involved in novel object exploration.

It is important to point out that in the present study we assumed that the activity of a cell assembly could be described as a linear combination of the activity of individual neurons. While this simplification of the assembly model allows for the analysis of large neuronal populations, it also presents some potential caveats (Lopes-dos-Santos et al., 2013). In particular, strong non-linear correlations between neurons may lead to spurious results, since both the determination of the number of assemblies and the extraction of assembly patterns are based on the linear model. Nevertheless, because this representation of assemblies is intuitive and straightforward, it is possible to verify the outcomes of the analysis; for instance, visual inspection of the raw data confirms that co-activations of assembly members correspond to peaks in assembly activity (see Figure 2B, also see examples employing similar linear methods in (Nicolelis et al., 1995; Peyrache et al., 2009, 2010; Benchenane et al., 2010; Lopes-dos-Santos et al., 2011, 2013). In principle, a non-linear method should be more robust and realistic, but we are not aware of any non-linear method capable of extracting assembly composition from the ongoing activity of neuronal populations with dozens of neurons. An ideal method should also incorporate information on the physiology of specific cell types and neural circuits. Taken together, our results show that, despite any possible non-linear correlations that may exist among neurons, the linear ones carry relevant information that support a role for phase sequences in behavior and cognition. Future research shall include non-linear modeling and also consider a neural coding approach, in order to fully characterize the repertoires of phase sequences, and elucidate the role of specific graph attributes in the representation of contextual cues, sensory stimuli and motor behavior.

## Author contributions

Sidarta Ribeiro collected the data; Daniel G. Almeida-Filho, Nivaldo A. P. Vasconcelos, Vitor Lopes-dos-Santos, and Sidarta Ribeiro analyzed the data; Daniel G. Almeida-Filho prepared the figures; Daniel G. Almeida-Filho, Sidarta Ribeiro, Nivaldo A. P. Vasconcelos, Vitor Lopes-dos-Santos, Adriano B. L.Tort, and José G. V. Miranda wrote the manuscript.

### Conflict of interest statement

The authors declare that the research was conducted in the absence of any commercial or financial relationships that could be construed as a potential conflict of interest.

## References

[B1] Adrian E. D. Zotterman Y. (1926). The impulses produced by sensory nerve-endings Part II. The response of a Single End-Organ. J. Physiol. 61, 151–171 16993780 10.1113/jphysiol.1926.sp002281 PMC1514782

[B2] Benchenane K. Peyrache A. Khamassi M. Tierney P. L. Gioanni Y. Battaglia F. P. (2010). Coherent theta oscillations and reorganization of spike timing in the hippocampal-prefrontal network upon learning. Neuron 66, 921–936 10.1016/j.neuron.2010.05.013 20620877

[B3] Berger D. Borgelt C. Louis S. Morrison A. Grün S. (2010). Efficient identification of assembly neurons within massively parallel spike trains. Comput. Intell. Neurosci. 2010, 1 10.1155/2010/439648 19809521 PMC2754663

[B4] Bi G.-Q. Poo M.-M. (1998). Synaptic modifications in cultured hippocampal neurons: dependence on spike timing, synaptic strength, and postsynaptic cell type. J. Neurosci. 18, 10464–10472 9852584 10.1523/JNEUROSCI.18-24-10464.1998 PMC6793365

[B5] Bliss T. V. Collingridge G. L. (1993). A synaptic model of memory: long-term potentiation in the hippocampus. Nature 361, 31–39 10.1038/361031a0 8421494

[B6] Buzsáki G. (2004). Large-scale recording of neuronal ensembles. Nat. Neurosci. 7, 446–451 10.1038/nn1233 15114356

[B7] Canolty R. T. Ganguly K. Kennerley S. W. Cadieu C. F. Koepsell K. Wallis J. D. (2010). Oscillatory phase coupling coordinates anatomically dispersed functional cell assemblies. Proc. Natl. Acad. Sci. U.S.A. 107, 17356–17361 10.1073/pnas.1008306107 20855620 PMC2951408

[B8] Chang F.-L. F. Isaacs K. R. Greenough W. T. (1991). Synapse formation occurs in association with the induction of long-term potentiation in two-year-old rat hippocampus *in vitro*. Neurobiol. Aging 12, 517–522 10.1016/0197-4580(91)90082-U 1770987

[B9] Deisseroth K. Bito H. Schulman H. Tsien R. (1995). Synaptic plasticity: a molecular mechanism for metaplasticity. Curr. Biol. 5, 1334–1338 10.1016/S0960-9822(95)00262-4 8749377

[B11] Denker M. Riehle A. Diesmann M. Grün S. (2010). Estimating the contribution of assembly activity to cortical dynamics from spike and population measures. J. Comput. Neurosci. 29, 599–613 10.1007/s10827-010-0241-8 20480218 PMC2978895

[B12] Dragoi G. Tonegawa S. (2010). Preplay of future place cell sequences by hippocampal cellular assemblies. Nature 469, 397–401 10.1038/nature09633 21179088 PMC3104398

[B13] Gervasoni D. Lin S. C. Ribeiro S. Soares E. S. Pantoja J. Nicolelis M. A. L. (2004). Global forebrain dynamics predict rat behavioral states and their transitions. J. Neurosci. 24, 11137–11147 10.1523/JNEUROSCI.3524-04.2004 15590930 PMC6730270

[B14] Harris K. D. (2005). Neural signatures of cell assembly organization. Nat. Rev. Neurosci. 6, 399–407 10.1038/nrn1669 15861182

[B15] Harris K. D. Csicsvari J. Hirase H. Dragoi G. Buzsáki G. (2003). Organization of cell assemblies in the hippocampus. Nature 424, 552–556 10.1038/nature01834 12891358

[B16] Hebb D. (1949). The Organization of Behavior. New York, NY: Wiley

[B17] Hobson J. A. McCarley R. W. (1971). Cortical unit activity in sleep and waking. Electroencephalogr. Clin. Neurophysiol. 30, 97–112 10.1016/0013-4694(71)90271-9 4100287

[B18] Hubel D. H. Wiesel T. N. (1959). Receptive fields of single neurones in the cat's striate cortex. J. Physiol. 148, 574–591 14403679 10.1113/jphysiol.1959.sp006308 PMC1363130

[B19] Hyvärinen A. Oja E. (2000). Independent component analysis: algorithms and applications. Neural Netw. 13, 411–430 10.1016/S0893-6080(00)00026-5 10946390

[B20] Ikegaya Y. Aaron G. Cossart R. Aronov D. Lampl I. Ferster D. (2004). Synfire chains and cortical songs: temporal modules of cortical activity. Science 304, 559–564 10.1126/science.1093173 15105494

[B21] Ji D. Wilson M. A. (2006). Coordinated memory replay in the visual cortex and hippocampus during sleep. Nat. Neurosci. 10, 100–107 10.1038/nn1825 17173043

[B22] John G. H. Langley P. (1995). Estimating continuous distributions in Bayesian classifiers, in Proceedings of the Eleventh conference on Uncertainty in artificial intelligence (San Francisco, CA: Morgan Kaufmann Publishers Inc.), 338–345

[B23] Klintsova A. Y. Greenough W. T. (1999). Synaptic plasticity in cortical systems. Curr. Opin. Neurobiol. 9, 203–208 10.1016/S0959-4388(99)80028-2 10322189

[B24] Kraus B. J. Robinson Ii R. J. White J. A. Eichenbaum H. Hasselmo M. E. (2013). Hippocampal time cells: time versus path integration. Neuron 78, 1090–1101 10.1016/j.neuron.2013.04.015 23707613 PMC3913731

[B25] Laubach M. Shuler M. Nicolelis M. A. (1999). Independent component analyses for quantifying neuronal ensemble interactions. J. Neurosci. Methods 94, 141–154 10.1016/S0165-0270(99)00131-4 10638821

[B26] Lee A. K. Wilson M. A. (2002). Memory of sequential experience in the hippocampus during slow wave sleep. Neuron 36, 1183–1194 10.1016/S0896-6273(02)01096-6 12495631

[B27] Liu X. Ramirez S. Pang P. T. Puryear C. B. Govindarajan A. Deisseroth K. (2012). Optogenetic stimulation of a hippocampal engram activates fear memory recall. Nature 484, 381–385 10.1038/nature11028 22441246 PMC3331914

[B28] Lopes-dos-Santos V. Conde-Ocazionez S. Nicolelis M. A. L. Ribeiro S. T. Tort A. B. L. (2011). Neuronal assembly detection and cell membership specification by principal component analysis. PLoS ONE 6:e20996 10.1371/journal.pone.0020996 21698248 PMC3115970

[B29] Lopes-dos-Santos V. Ribeiro S. Tort A. B. (2013). Detecting cell assemblies in large neuronal populations. J. Neurosci. Methods 220, 149–166 10.1016/j.jneumeth.2013.04.010 23639919

[B30] Macdonald C. J. Lepage K. Q. Eden U. T. Eichenbaum H. (2011). Hippocampal time cells bridge the gap in memory for discontiguous events. Neuron 71, 737–749 10.1016/j.neuron.2011.07.012 21867888 PMC3163062

[B31] Marčenko V. A. Pastur L. A. (1967). Distribution of eigenvalues for some sets of random matrices. Mat. Sb. 1, 457–483 10.1070/SM1967v001n04ABEH001994

[B32] Moritz C. T. Perlmutter S. I. Fetz E. E. (2008). Direct control of paralysed muscles by cortical neurons. Nature 456, 639–642 10.1038/nature07418 18923392 PMC3159518

[B33] Mota N. B. Vasconcelos N. A. P. Lemos N. Pieretti A. C. Kinouchi O. Cecchi G. A. (2012). Speech graphs provide a quantitative measure of thought disorder in psychosis. PLoS ONE 7:e34928 10.1371/journal.pone.0034928 22506057 PMC3322168

[B34] Nicolelis M. A. Baccala L. A. Lin R. Chapin J. K. (1995). Sensorimotor encoding by synchronous neural ensemble activity at multiple levels of the somatosensory system. Science 268, 1353–1358 10.1126/science.7761855 7761855

[B35] Nicolelis M. A. L. Dimitrov D. Carmena J. M. Crist R. Lehew G. Kralik J. D. (2003). Chronic, multisite, multielectrode recordings in macaque monkeys. Proc. Natl. Acad. Sci. U.S.A. 100, 11041–11046 10.1073/pnas.1934665100 12960378 PMC196923

[B36] Noda H. Manohar S. Adey W. R. (1969). Spontaneous activity of cat hippocampal neurons in sleep and wakefulness. Exp. Neurol. 24, 217–231 10.1016/0014-4886(69)90016-8 4306533

[B37] O'Keefe J. Dostrovsky J. (1971). The hippocampus as a spatial map. Preliminary evidence from unit activity in the freely-moving rat. Brain Res. 34, 171–175 10.1016/0006-8993(71)90358-1 5124915

[B38] Pastalkova E. Itskov V. Amarasingham A. Buzsáki G. (2008). Internally generated cell assembly sequences in the rat hippocampus. Science 321, 1322–1327 10.1126/science.1159775 18772431 PMC2570043

[B38a] Paxinos G. Watson C. (1997). The Rat Brain in Stereotaxic Coordinates. San Diego, CA: Academic Press

[B39] Peyrache A. Benchenane K. Khamassi M. Wiener S. I. Battaglia F. P. (2010). Principal component analysis of ensemble recordings reveals cell assemblies at high temporal resolution. J. Comp. Neurosci. 29, 309–325 10.1007/s10827-009-0154-6 19529888 PMC2940043

[B40] Peyrache A. Khamassi M. Benchenane K. Wiener S. I. Battaglia F. P. (2009). Replay of rule-learning related neural patterns in the prefrontal cortex during sleep. Nat. Neurosci. 12, 919–926 10.1038/nn.2337 19483687

[B41] Pfeiffer B. E. Foster D. J. (2013). Hippocampal place-cell sequences depict future paths to remembered goals. Nature 497, 74–79 10.1038/nature12112 23594744 PMC3990408

[B42] Ramirez S. Liu X. Lin P. A. Suh J. Pignatelli M. Redondo R. L. (2013). Creating a false memory in the hippocampus. Science 341, 387–391 10.1126/science.1239073 23888038

[B43] Ribeiro S. Shi X. Engelhard M. Zhou Y. Zhang H. Gervasoni D. (2007). Novel experience induces persistent sleep-dependent plasticity in the cortex but not in the hippocampus. Front. Neurosci. 1:43 10.3389/neuro.01.1.1.003.2007 18982118 PMC2577304

[B44] Robbe D. Montgomery S. M. Thome A. Rueda-Orozco P. E. Mcnaughton B. L. Buzsaki G. (2006). Cannabinoids reveal importance of spike timing coordination in hippocampal function. Nat. Neurosci. 9, 1526–1533 10.1038/nn1801 17115043

[B45] Schrader S. Grün S. Diesmann M. Gerstein G. L. (2008). Detecting synfire chain activity using massively parallel spike train recording. J. Neurophysiol. 100, 2165–2176 10.1152/jn.01245.2007 18632888 PMC2576207

[B46] Stopfer M. Bhagavan S. Smith B. H. Laurent G. (1997). Impaired odour discrimination on desynchronization of odour-encoding neural assemblies. Nature 390, 70–74 10.1038/36335 9363891

[B47] Vanderwolf C. (1969). Hippocampal electrical activity and voluntary movement in the rat. Electroencephalogr. Clin. Neurophysiol. 26, 407–418 10.1016/0013-4694(69)90092-3 4183562

[B48] Wilson M. A. McNaughton B. L. (1994). Reactivation of hippocampal ensemble memories during sleep. Science 265, 676–679 10.1126/science.8036517 8036517

